# Impact of Implementing CYP2C19 Genotype-Guided Antiplatelet Therapy on P2Y_12_ Inhibitor Selection and Clinical Outcomes in Acute Coronary Syndrome Patients After Percutaneous Coronary Intervention: A Real-World Study in China

**DOI:** 10.3389/fphar.2020.582929

**Published:** 2021-01-20

**Authors:** Yi Zhang, Xiu-Jin Shi, Wen-Xing Peng, Jia-Lun Han, Bai-Di Lin, Ru Zhang, Yun-Nan Zhang, Jia-Lin Yan, Juan-Juan Wei, Yi-Fan Wang, Su-Wei Chen, Nan Nan, Zhen-Wei Fang, Yong Zeng, Yang Lin

**Affiliations:** ^1^ Department of Pharmacy, Beijing Anzhen Hospital, Capital Medical University, Beijing, China; ^2^ Department of Cardiovascular Surgery, Beijing Anzhen Hospital, Capital Medical University, Beijing, China; ^3^ Department of Cardiology, Beijing Anzhen Hospital, Capital Medical University, Beijing, China

**Keywords:** CYP2C19, clopidogrel, ticagrelor, acute coronary syndrome, gene-guided antiplatelet therapy

## Abstract

**Background:** CYP2C19 loss-of-function (LOF) alleles reduce the effectiveness of clopidogrel in patients undergoing percutaneous coronary intervention for acute coronary syndrome. However, the clinical impact of implementing CYP2C19 gene-guided pharmacotherapy is unclear, especially among the Chinese population. The purpose of this study was to evaluate P2Y12 receptor inhibitor selection and clinical outcomes upon implementation of CYP2C19 genotype-guided pharmacotherapy in current clinical practice.

**Methods:** This was a single-center observational cohort study. Adult percutaneous coronary intervention patients who received CYP2C19 genetic testing (*2, *3, *17 alleles) were included. Ticagrelor was recommended for patients with a LOF allele. Factors related to P2Y12 inhibitor selection were determined by logistic regression. The primary endpoint was major cardiac or cerebrovascular adverse events (MACCE) within 12 months. MACCE and clinically significant bleeding events (BARC ≥2) in the LOF-clopidogrel group, non-LOF-clopidogrel group, and non-LOF-ticagrelor group were compared with those in the LOF-ticagrelor group. The inverse probability of treatment weighting (IPTW) was adjusted in a Cox regression analysis to eliminate confounding factors.

**Results:** Among 1,361 patients, 826 (60.7%) had a LOF allele. Patients with a LOF allele were more likely to be prescribed ticagrelor (multivariate-adjusted OR 1.349; 95% CI 1.040 to 1.751; *p* = 0.024). The MACCE rate was higher in the LOF-clopidogrel group than in the LOF-ticagrelor group (7.8 vs. 4.0%; log-rank *p* = 0.029; IPTW-adjusted HR 2.138; 95% CI 1.300–3.515). Compared with the LOF-ticagrelor group, the non-LOF-clopidogrel group showed no significant difference in MACCE rate (5.8 vs. 4.0%; log-rank *p* = 0.272; IPTW-adjusted HR 1.531; 95% CI 0.864–2.714). Among the patients treated with ticagrelor, there was no significant difference in the MACCE rate between the LOF group and non-LOF group (4.3 vs. 4.0%; log-rank *p* = 0.846; IPTW-adjusted HR 1.184; 95% CI 0.582–2.410). There was no significant difference in the incidence of clinically significant bleeding events among the four groups.

**Conclusion:** This study confirms that efficiently returned CYP2C19 genotype results did partially guide cardiologists to prescribe ticagrelor for patients with a LOF allele, and that clopidogrel had a higher risk of MACCE than ticagrelor in these patients, which provides support for the implementation of CYP2C19 gene-guided antiplatelet therapy in clinical practice.

## Introduction

Coronary artery disease (CAD), specifically acute coronary syndrome (ACS), is still the leading cause of disability and mortality worldwide (Fox et al., 2010; Jernberg et al., 2015). Oral dual antiplatelet therapy with aspirin and a P2Y_12_ receptor inhibitor is the standard postoperative maintenance strategy for patients undergoing percutaneous coronary intervention (PCI) for ACS (Levine et al., 2011; Levine et al., 2016). Clopidogrel, the most widely used P2Y_12_ inhibitor, was previously shown to improve the prognosis of ACS patients. However, evidence from pharmacogenomics has gradually raised concerns about the clinical efficacy of clopidogrel. As a prodrug, clopidogrel needs to be converted into active substances by cytochrome (CYP2C19) enzymes. Patients with a CYP2C19 loss-of-function (LOF) allele were shown to have lower conversion rates of clopidogrel by their CYP2C19 enzymes (Mega et al., 2009; Shuldiner et al., 2009). The population frequency of a LOF allele is approximately 60% in East Asian countries and approximately 30% in the rest of the world (Klein et al., 2019). Our previous study identified possession of a CYP2C19 LOF allele is an independent risk factor for clopidogrel-related high platelet responsiveness (Peng et al., 2019), which reduces the effectiveness of the drug (Mega et al., 2009; Shuldiner et al., 2009; Mao et al., 2013; Xi et al., 2019).

Ticagrelor is a P2Y_12_ inhibitor that acts directly on platelets, and its efficacy is hardly affected by CYP2C19 gene polymorphisms (Wallentin et al., 2010; Li et al., 2017). The PLATO trial confirmed that ticagrelor is superior to clopidogrel in reducing adverse cardiovascular events in patients with ACS (Wallentin et al., 2009). Furthermore, current guidelines recommend the use of ticagrelor or prasugrel in preference to clopidogrel in ACS patients (Levine et al., 2011; Levine et al., 2016). However, the high incidences of bleeding and discontinuation due to dyspnea, as well as the high price, have limited the widespread clinical use of ticagrelor (Wallentin et al., 2009; Bonaca et al., 2015).

Emerging evidence from observational studies and randomized controlled trials has confirmed the feasibility of implementing CYP2C19 gene-guided pharmacotherapy (Cavallari et al., 2018b; Lee et al., 2018; Notarangelo et al., 2018; Claassens et al., 2019; Tuteja et al., 2020). In addition, increasing numbers of medical institutions are beginning to incorporate CYP2C19 genotyping into clinical practice. However, the PCI guidelines state that CYP2C19 pharmacogenetic testing should only be considered for high-risk patients (Class IIb; Level of Evidence: C) (Levine et al., 2011; Levine et al., 2016). In the absence of strong support from current guidelines, there are limited real-world data in East Asian populations to validate the implementation of genotype-guided antiplatelet therapy in clinical practice. Furthermore, the impact of genetic testing results on P2Y_12_ inhibitor selection by doctors in China remains unclear.

Therefore, we performed a single-center retrospective observational study in Chinese patients with ACS after PCI, with the following objectives: 1) to determine the effects of returned genotype results on the prescription behavior of cardiologists and 2) to understand the relationships of the identified genotypes with P2Y_12_ receptor inhibitor selection and clinical outcomes.

## Methods

### Patients and Study Design

PHARM-ACS is an ambispective single-center ongoing observational registry study on PHARMacotherapy and long-term clinical outcomes in patients with ACS after PCI, initiated by the Department of Pharmacy at Beijing Anzhen Hospital (NCT04184583). In this registry, consecutive adult patients in our hospital with successfully indexed PCI with indication of ST-segment elevation myocardial infarction (STEMI), non-STEMI, or unstable angina were eligible between April 2018 and December 2021. Patients were recruited retrospectively from April 2018 to September 2019 and prospectively from October 2019 to December 2021. Follow-up will be conducted until December 2023. All available clinical features, detailed medical history, medication information, genetics information, and clinical outcomes were retrospectively/prospectively documented in an electronic data capture system (EDCs) and regularly monitored for data quality. The Ethics Committee of the Clinical Research Center at Beijing Anzhen Hospital approved the research protocol. All registered patients signed informed consent.

Data for the present study were extracted for consecutive patients registered between April 2018 and December 2018 as part of the PHARM-ACS registry. The inclusion criteria were: 1) age ≥18 years and 2) detection of CYP2C19 genotype (*2, *3, *17 alleles) during hospitalization. The exclusion criteria were: 1) patients who were not prescribed a P2Y_12_ inhibitor at discharge, 2) patients who were prescribed cilostazol at discharge, 3) patients who could not cooperate with the research protocol or withdraw informed consent, and 4) patients with in-hospital death that was not due to stent thrombosis.

### CYP2C19 Genotyping and Phenotyping

CYP2C19 genotype testing (*2, *3, *17 alleles) was performed at the Center for Clinical and Pharmaceutical Precision Testing, Department of Pharmacy, Beijing Anzhen Hospital. Genomic DNA was extracted from leukocytes of peripheral blood and stored in 3 ml ethylenediaminetetraacetic acid-anticoagulated vacuum tubes. CYP2C19 genotypes were determined by fluorescence *in situ* hybridization (TL988A, Xi'an TianLong, Xi'an, China) including the following variant alleles: CYP2C19∗2 (rs4244285), CYP2C19∗3 (rs4986893), and CYP2C19∗17 (rs12248560). The whole process was performed according to the manufacturer's instructions. According to the recommendations of the Clinical Pharmacogenetics Implementation Consortium (CPIC) (Scott et al., 2013), the phenotypes of CYP2C19 were divided into LOF (IM/PM; IM: intermediate metabolizer,*1/*2, *1/*3, *2/*17, or *3/*17; PM: poor metabolizer,*2/*2, *2/*3, or *3/*3) and non-LOF (UM/RM/NM; UM: ultra-rapid metabolizer, *17/*17; RM: rapid metabolizer, *1/*17; NM: normal metabolizer, *1/*1).

After obtaining informed consent from the patient, and within 48 h after the order from their doctor came into effect, a genotype test report with drug selection recommendations was reviewed by the pharmacist and returned to the clinician via the electronic medical record system (EMRs). Although ticagrelor was recommended for patients with PM/IM, the clinical characteristics (such as ischemia or bleeding risk factors) and economic conditions of patients were also considered by cardiologists when selecting P2Y_12_ inhibitors. The P2Y_12_ inhibitor prescribed on discharge was at the discretion of the cardiologist. Because prasugrel is not licensed in China, the maintenance of antiplatelet therapy for ACS patients after standard PCI procedures was recommended as aspirin (100 mg/day) combined with clopidogrel (75 mg/day) or ticagrelor (90 mg/twice a day) for at least 12 months. Prescription of ticagrelor for IM/PM patients at discharge was considered to comply with the genotype-guided therapy.

### Follow-Up and Endpoints

All patients had scheduled visits at 6 and 12 months, and annually thereafter. During the examinations, the patients were asked to fill out questionnaires by trained followers. The questionnaire was designed by the researcher team and involved the following content: demographic information, medication information, medication compliance, adverse drug reactions, clinical events and quality of life. Medication compliance was evaluated with the 8-item Morisky medication adherence scale (MMAS-8) score (Krousel-Wood et al., 2009). Information on any outcome event in patients was obtained by the investigator via electronic medical records, telephone, or WeChat, and uploaded to the EDCs.

The primary endpoint was a composite of major adverse cardiovascular or cerebrovascular events (MACCE), including all-cause death, stent thrombosis, stroke, myocardial infarction, and any urgent coronary revascularization within 1 year after the indexed PCI. The secondary endpoint was a composite of MACCE plus unstable angina within 12 months of follow-up. The safety endpoint was clinically significant bleeding events, defined as Bleeding Academic Research Consortium (BARC) class 2 or higher bleeding events within 12 months. All bleeding events were defined according to the BARC criteria (Ndrepepa et al., 2012). BARC class 2 was considered a moderate bleeding event, and BARC class 3 or higher was considered a severe bleeding event. Identification of a MACCE was based on diagnostic records derived from the EMRs or diagnostic reports provided by patients. Self-reported information from patients was adopted to assist in the identification of bleeding events and unstable angina. All clinical events were verified by at least two cardiologists.

## Statistical Analysis

Baseline demographic and clinical factors were assessed using descriptive statistics. Frequency (percentage) was used to report discrete variables. Mean ± standard deviation was used to report continuous variables. Unadjusted comparisons between groups were performed using Fisher’s exact test, chi-square test, or Student’s *t*-test, as appropriate. Demographic and clinical factors related to P2Y_12_ inhibitor selection were determined by univariate and multivariate logistic regression. Patients with no events within 1 year after PCI were censored at the time of the most recent follow-up. The 1-year cumulative event rates of MACCE, MACCE plus unstable angina, and clinically significant bleeding events were plotted using the Kaplan-Meier method and evaluated by the log-rank test. Survival analysis was implemented using Cox proportional hazards models. Three pairs of comparisons were constructed separately, comprising LOF-clopidogrel vs. LOF-ticagrelor, non-LOF-clopidogrel vs. LOF- ticagrelor, and non-LOF-ticagrelor vs. LOF- ticagrelor. Inverse probability treatment weighting (IPTW) was performed to avoid bias in comparisons. Differences between groups were examined with standardized differences using a 10% threshold to indicate significant meaningful imbalances in covariates (Austin, 2015). Three sensitivity analyses and secondary analyses were conducted.

Kaplan–Meier curves were drawn using Prism 7.0 (GraphPad Software, La Jolla, CA). Statistical analyses were performed using R software version 3.4.3 (https://www.r-project.org/). Values of *p* < 0.05 were considered statistically significant.

## Results

**FIGURE 1:**
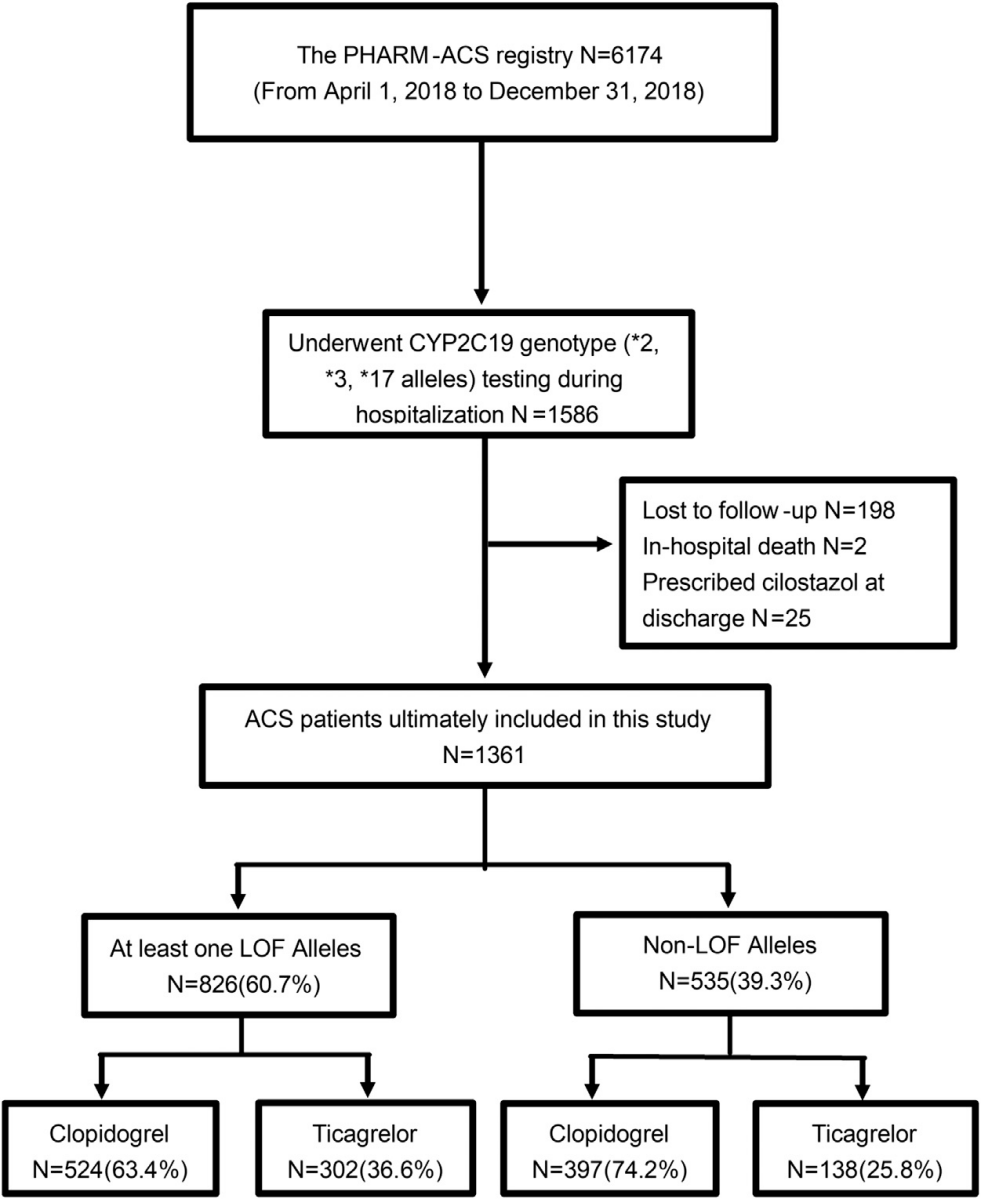
Flowchart of cohort selection.

The present study included 1,361 patients who underwent PCI for ACS (Figure 1). The mean age was 60.16 ± 9.73 years, 24.32% were female, 98.02% were Han nationality, and 92.29% had an implanted stent. Comorbidities such as hypertension (63.56%), hyperlipidemia (52.31%), and diabetes mellitus (34.53%) were common. Before admission, 26.97% of patients were on P2Y_12_ inhibitors.

The baseline demographic and clinical factors in the LOF-clopidogrel group, non-LOF-clopidogrel group, and non-LOF-ticagrelor group were compared with those in the LOF-ticagrelor group (Table 1). As shown in Table 2, after the values were well-balanced by IPTW adjustment, the differences between the LOF-clopidogrel group and LOF-ticagrelor group were anticoagulant agent use and end-stage kidney disease. The imbalances between the non-LOF-clopidogrel group and LOF-ticagrelor group were ticagrelor use before admission, anticoagulant agent use, and end-stage kidney disease. End-stage kidney disease differed between the non-LOF-ticagrelor group and LOF-ticagrelor group.

**TABLE 1:** Baseline demographic and clinical factors.

	All patients	LOF-clopidogrel	LOF-ticagrelor	Non-LOF-clopidogrel	Non-LOF-ticagrelor
	*n* = 1,361 (%)	*n* = 524 (%)	*n* = 302 (%)	*n* = 397 (%)	*n* = 138 (%)
Age, years	60.16 ± 9.73	60.98 ± 10.03^a^	58.60 ± 9.30	60.78 ± 9.50^a^	58.70 ± 9.72
≥75	91 (6.7)	43 (8.2)^a^	11 (3.6)	32 (8.1)^a^	5 (3.6)
Female	331 (24.3)	141 (26.9)	65 (21.5)	97 (24.4)	28 (20.3)
Ethnic					
Han	1,334 (98.0)	515 (98.3)	294 (97.4)	392 (98.7)	133 (96.4)
minorities	27 (2.0)	9 (1.7)	8 (2.7)	5 (1.3)	5 (3.6)
BMI, kg/m^2^ ^b^	25.83 ± 3.29	25.66 ± 3.24^a^	26.15 ± 3.47	25.85 ± 3.24	25.67 ± 3.18
>30	149 (11.2)	56 (11.0)	34 (11.3)	42 (10.8)	17 (12.5)
Current smoker	303 (22.3)	110 (21.0)	78 (25.8)	90 (22.7)	25 (18.1)
PCI indicatin					
STEMI	83 (6.1)	27 (5.2)^a^	27 (8.9)	15 (3.8)^a^	14 (10.1)
Non-STEMI	93 (6.8)	31 (5.9)	27 (8.9)	23 (5.8)	12 (8.7)
Unstable angina	1,185 (87.1)	466 (88.9)^a^	248 (82.1)	359 (90.4)^a^	112 (81.2)
No. of stent					
<1	105 (7.7)	48 (9.2)^a^	13 (4.3)	40 (10.1)^a^	4 (2.9)
≥1, <3	1,029 (75.6)	400 (76.3)	226 (74.8)	297 (74.8)	106 (76.8)
≥3	227 (16.7)	76 (14.5)^a^	63 (20.9)	60 (15.1)^a^	28 (20.3)
P2y12 inhibitor use before admission					
Clopidogrel	331 (24.3)	149 (28.4)^a^	53 (17.6)	99 (24.9)^a^	30 (21.7)
Ticagrelor	36 (2.7)	3 (0.6)^a^	20 (6.6)	2 (0.5)^a^	11 (8.0)
Medical history					
Previous PCI with stenting	434 (31.9)	171 (32.6)	100 (33.1)	122 (30.7)	41 (29.7)
Previous CABG	54 (4.0)	20 (3.8)	12 (4.0)	18 (4.5)	4 (2.9)
Hypertension	865 (63.6)	350 (66.8)^a^	170 (56.3)	260 (65.5)^a^	85 (61.6)
Hyperlipidemia	712 (52.3)	266 (50.8)	160 (53.0)	207 (52.1)	79 (57.3)
Diabetes mellitus	470 (34.5)	183 (34.9)	97 (32.1)	130 (32.8)	60 (43.5)^a^
End-stage kidney disease	18 (1.3)	10 (1.9)	1 (0.3)	4 (1.0)	3 (2.2)
Myocardial infarction	184 (13.5)	62 (11.8)	46 (15.2)	56 (14.1)	20 (14.5)
Atrial fibrillation	35 (2.6)	16 (3.1)	2 (0.7)	15 (3.8)	2 (1.5)
Heart failure	16 (1.2)	9 (1.7)	2 (0.7)	4 (1.0)	1 (0.7)
Gastrointestinal bleed	3 (0.2)	2 (0.4)	0 (0.0)	1 (0.3)	0 (0.0)
Cerebral infarction	97 (7.1)	46 (8.8)	18 (6.0)	26 (6.6)	7 (5.1)
Left ventricular EF%^b^	62.78 ± 7.31	63.20 ± 7.43	62.13 ± 7.71	63.06 ± 6.98	61.95 ± 6.84
eGFR^c^	0.57 ± 0.13	0.57 ± 0.14	0.57 ± 0.13	0.57 ± 0.12	0.59 ± 0.14
Discharge medication					
Aspirin	1,357 (99.7)	521 (99.4)	301 (99.7)	397 (100.0)	138 (100.0)
Anticoagulant agent	10 (0.7)	6 (1.2)	1 (0.3)	3 (0.8)	0 (0.0)
Statin	1,350 (99.2)	518 (98.9)	301 (99.7)	394 (99.2)	137 (99.3)
ACEI or ARB	457 (33.6)	181 (34.5)	91 (30.1)	139 (35.0)	46 (33.3)
Beta blocker	918 (67.5)	359 (68.5)	207 (68.5)	263 (66.3)	89 (64.5)
Proton pump inhibitor	1,192 (87.6)	445 (84.9)^a^	277 (91.7)	349 (87.9)	121 (87.7)
Morisky score ≥6^d^	1,313 (96.5)	509 (97.1)	290 (96.0)	383 (96.5)	131 (94.9)

Values are mean ± SD or *n* (%).

LOF: loss-of-function; BMI: body mass index; PCI: percutaneous coronary intervention; CABG: coronary-artery bypass grafting; STEMI: ST-segment elevation myocardial infarction; NSTEMI: non-ST-segment elevation myocardial infarction; EF: ejection fraction; eGFR: estimate glomerular filtration rate; ACEI: angiotensin-converting enzyme inhibitor; ARB: angiotensin receptor blocker.

^a^ p < 0.05 compared with LOF-ticagrelor group.

^b^ There are 23 missing data in BMI and 172 missing data in Left ventricular EF.

^c^ The eGFR value is calculated based on the MDRD formula, and there were 12 patients with missing data.

^d^ The Morisky score was examined at the most recent follow-up. A score ≥6 was determined to have good medication compliance.

**TABLE 2:** Patient demographic and clinical factors after adjustment with inverse probability of treatment weights (IPTW).

	LOF-ticagrelor	LOF-clopidogrel	IPTW-adjusted standardized difference	LOF-ticagrelor	Non-LOF-clopidogrel	IPTW-adjusted standardized difference	LOF-ticagrelor	Non-LOF-ticagrelor	IPTW-adjusted standardized difference
	*n* = 302 (%)	*n* = 524 (%)		*n* = 302 (%)	*n* = 397 (%)		*n* = 302 (%)	*n* = 138 (%)	
Age	59.67 ± 9.01	60.20 ± 10.59	0.05	59.74 ± 9.16	59.99 ± 10.00	0.03	58.79 ± 9.31	59.00 ± 9.66	0.02
Female	75 (25.0)	127 (24.2)	0.02	74 (24.6)	97 (24.4)	0.01	63 (20.8)	27 (19.9)	0.02
Han	296 (97.9)	511 (97.6)	0.02	296 (97.9)	388 (97.8)	0.00	294 (97.2)	134 (97.1)	0.00
Body mass index	25.78 ± 3.33	25.81 ± 3.31	0.01	25.91 ± 3.37	25.92 ± 3.03	0.00	25.95 ± 3.41	25.90 ± 3.20	0.02
Current smoker	68 (22.5)	117 (22.4)	0.00	73 (24.1)	92 (23.1)	0.02	70 (23.1)	33 (23.8)	0.02
PCI indicatin									
STEMI	21 (7.0)	36 (6.9)	0.00	20 (6.7)	24 (6.1)	0.02	29 (9.5)	13 (9.4)	0.00
Non-STEMI	19 (6.3)	31 (5.9)	0.02	22 (7.2)	27 (6.7)	0.02	28 (9.2)	13 (9.4)	0.01
No. of stent									
<1	23 (7.7)	42 (8.1)	0.01	27 (8.8)	32 (8.1)	0.03	12 (3.9)	5 (3.9)	0.00
≥1, <3	222 (73.6)	392 (74.7)	0.03	224 (74.0)	297 (74.9)	0.02	226 (75.0)	105 (75.8)	0.02
≥3	56 (18.7)	90 (17.2)	0.04	52 (17.2)	68 (17.0)	0.00	64 (21.2)	28 (20.4)	0.02
P2Y_12_ inhibitor use before admission									
Clopidogrel	81 (26.9)	136 (25.9)	0.02	69 (22.8)	93 (23.3)	0.01	58 (19.3)	27 (19.6)	0.01
Ticagrelor	10 (3.2)	11 (2.1)	0.07	10 (3.4)	5 (1.2)	**0.15**	22 (7.3)	11 (7.9)	0.02
Medical history									
Previous PCI with stenting	98 (32.4)	171 (32.7)	0.01	104 (34.5)	133 (33.6)	0.02	96 (31.8)	45 (32.3)	0.01
Previous CABG	10 (3.3)	18 (3.5)	0.01	11 (3.8)	16 (4.0)	0.01	10 (3.3)	4 (3.2)	0.01
Hypertension	187 (62.0)	324 (61.9)	0.00	185 (61.3)	244 (61.6)	0.01	171 (56.7)	80 (57.8)	0.02
Hyperlipidemia	169 (56.1)	283 (54.0)	0.04	167 (55.3)	219 (55.1)	0.00	172 (56.9)	78 (56.7)	0.00
Diabetes mellitus	98 (32.6)	172 (32.7)	0.00	91 (30.1)	123 (31.0)	0.02	106 (35.2)	50 (36.0)	0.02
Myocardial infarction	36 (12.0)	63 (12.0)	0.00	50 (16.4)	59 (14.9)	0.04	42 (13.9)	20 (14.6)	0.02
Atrial fibrillation	7 (2.3)	12 (2.2)	0.01	8 (2.8)	12 (3.0)	0.01	2 (0.8)	1 (0.7)	0.00
Gastrointestinal bleed	0 (0.0)	0 (0.0)	0.00	0 (0.0)	1 (0.3)	0.08	0 (0.0)	0 (0.0)	0.00
End-stage kidney disease	0 (0.0)	5 (1.0)	**0.14**	0 (0.0)	3 (0.7)	**0.12**	0 (0.0)	1 (0.8)	**0.12**
Heart failure	4 (1.2)	6 (1.1)	0.01	3 (1.0)	4 (1.1)	0.01	2 (0.8)	1 (0.8)	0.00
Cerebral infarction	25 (8.3)	39 (7.5)	0.03	24 (8.1)	27 (6.8)	0.05	17 (5.8)	8 (5.8)	0.00
Left ventricular EF%	62.93 ± 7.03	63.01 ± 7.65	0.01	62.61 ± 7.14	62.79 ± 7.19	0.03	62.27 ± 7.72	62.26 ± 6.72	0.00
eGFR	0.58 ± 0.13	0.57 ± 0.14	0.07	0.57 ± 0.13	0.57 ± 0.12	0.00	0.58 ± 0.13	0.58 ± 0.14	0.00
Discharge medication									
Aspirin	301 (99.7)	521 (99.4)	0.03	301 (99.7)	397 (100.0)	0.08	301 (99.6)	138 (100.0)	0.09
Anticoagulant agent	0 (0.0)	4 (0.7)	**0.12**	0 (0.0)	2 (0.5)	**0.10**	0 (0.0)	0 (0.0)	—
Statin	300 (99.4)	520 (99.3)	0.01	301 (99.6)	394 (99.3)	0.03	301 (99.6)	137 (99.6)	0.00
ACEI or ARB	98 (32.5)	167 (31.9)	0.01	98 (32.6)	133 (33.6)	0.02	95 (31.3)	43 (31.3)	0.00
Beta blocker	202 (66.8)	354 (67.5)	0.02	198 (65.7)	263 (66.2)	0.01	201 (66.4)	91 (65.8)	0.01
Proton pump inhibitor	263 (87.2)	459 (87.7)	0.01	274 (90.8)	357 (89.9)	0.03	274 (90.8)	125 (90.6)	0.01
Morisky score ≥6	292 (96.7)	506 (96.6)	0.00	290 (96.2)	382 (96.2)	0.00	289 (95.6)	132 (95.9)	0.01

Values are mean ± SD or *n* (%).

If the Standardized Difference ≥0.10, it indicates that the variable is not well balanced between groups after adjusting IPTW. LOF, loss-of-function; PCI, percutaneous coronary intervention; CABG, coronary-artery bypass grafting; STEMI, ST-segment elevation myocardial infarction; NSTEMI, non-ST-segment elevation myocardial infarction; EF, ejection fraction; eGFR, estimate glomerular filtration rate; ACEI, angiotensin-converting enzyme inhibitor; ARB, angiotensin receptor blocker.

### CYP2C19 Genotypes and P2Y_12_ Inhibitor Selection

An overview of the genotype results is provided in Supplementary Figure S1. All genotype results were available before the patients were discharged. Among the total included patients, 826 (60.7%) carried at least one CYP2C19 LOF allele [IM: 660 (48.5%); PM: 166 (12.2%)], and no UM patients were detected. Clopidogrel (67.7%) was the most frequently used P2Y_12_ inhibitor, even in patients with a LOF allele (63.4%). Compared with non-LOF patients, ticagrelor was prescribed more frequently in IM/PM patients (36.6 vs. 25.8%) (Figure 1). Furthermore, 44.0 and 34.7% of PMs and IMs were prescribed ticagrelor for maintenance therapy (Supplementary Figure S2).

The clinical factors related to the P2Y_12_ inhibitor selection are shown in Supplementary Table S1. No high-dose clopidogrel prescription was observed. Patients with a LOF allele were more likely to be prescribed ticagrelor (multivariate-adjusted OR 1.349; 95% CI 1.040–1.751; *p* = 0.024). Clinical factors of ticagrelor use before admission (OR 16.850; 95% CI 5.845–48.573; *p* < 0.001), stent implantation (OR 2.515; 95% CI 1.414–4.473; *p* = 0.002), and number of stents implanted (OR 1.261; 95% CI 1.119–1.421; *p* < 0.001) were significantly associated with selection of ticagrelor. The clinical factors associated with clopidogrel selection were elderly age (OR 0.984; 95% CI 0.970–0.997; *p* = 0.018), hypertension (OR 0.673; 95% CI, 0.519–0.872; *p* = 0.003), indexed PCI for unstable angina (OR 0.58; 95% CI, 0.40–0.84; *p* = 0.004), and clopidogrel use before admission (OR 0.591; 95% CI 0.437–0.800; *p* < 0.001).

### Clinical Outcomes

**FIGURE 2:**
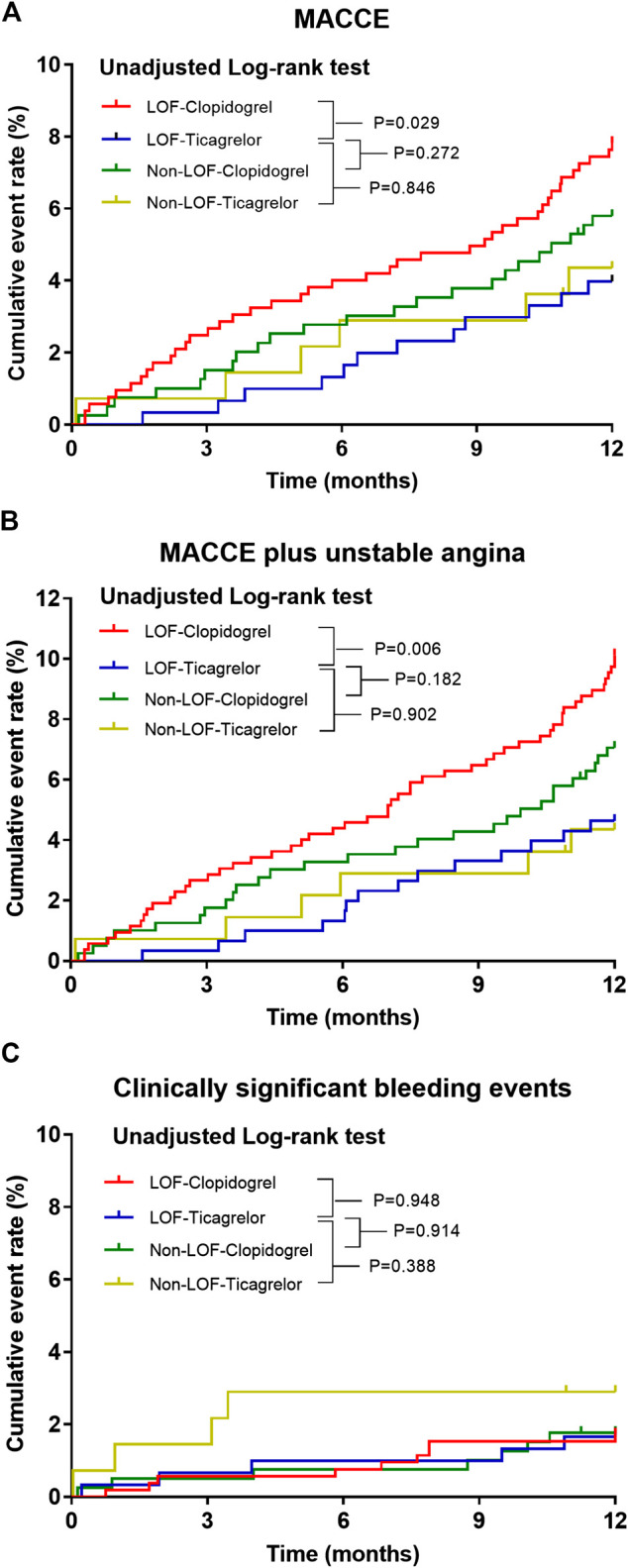
Kaplan–Meier estimate of cardiovascular or bleeding events according to CYP2C19 status and P2Y_12_ inhibitor during 12 months follow-up. Kaplan–Meier curves for **(A)** primary endpoints, **(B)** secondary endpoints, and **(C)** clinically significant bleeding event incidence in ACS patients post-PCI.

The median time from indexed PCI to MACCE or last follow-up was 15.6 (14.5–17.4) months. During the 12-months follow-up period after PCI, a total of 82 (6.0%) patients experienced MACCE, defined as a composite of death, myocardial infarction, stroke, urgent need for revascularization, and stent thrombosis. Clinically significant bleeding events occurred in 25 (1.8%) patients. The distributions of specific clinical events are shown in Table 3. The MACCE rate was higher in the LOF-clopidogrel group compared with the LOF-ticagrelor group (7.8 vs. 4.0%; log-rank *p* = 0.029; IPTW-adjusted HR, 2.138; 95% CI, 1.300–3.515). Compared with the LOF-ticagrelor group, the non-LOF-clopidogrel group showed no significant difference in the incidence of MACCE (5.8 vs. 4.0%; log-rank *p* = 0.272; IPTW-adjusted HR, 1.531; 95% CI, 0.864–2.714). Among the patients treated with ticagrelor, there was no significant difference in the event rate between the non-LOF group and LOF group (4.3 vs. 4.0%; log-rank *p* = 0.846; IPTW-adjusted HR, 1.184; 95% CI, 0.582–2.410) (Figure 2A and Table 4). We also compared the incidence of MACCE between the non-LOF-ticagrelor group and non-LOF-clopidogrel group, and no significant difference was observed (Supplementary Tables S2 and S3). Consistent with the analysis of the overall population, we observed that LOF-ticagrelor was superior to LOF-clopidogrel in reducing the risk of MACCE in the subset of patients with unstable angina (IPTW-adjusted HR 2.051; 95% CI 1.213–3.467, *p* = 0.007) (Supplementary Table S4).

**TABLE 3:** Cardiovascular and Bleeding Event Type within 12 Months by CYP2C19 Status and P2Y_12_ inhibitors.

	All patients	LOF-clopidogrel	LOF-ticagrelor	Non-LOF-clopidogrel	Non-LOF-ticagrelor
	*n* = 1,361	*n* = 524	*n* = 302	*n* = 397	*n* = 138
MACCE	82 (6.0)	41 (7.8)	12 (4.0)	23 (5.8)	6 (4.3)
Myocardial infarction	11 (0.8)	4 (0.8)	1 (0.3)	3 (0.8)	3 (2.2)
Stroke	12 (0.9)	6 (1.1)	1 (0.3)	5 (1.3)	0 (0.0)
Death	9 (0.7)	7 (1.3)	1 (0.3)	1 (0.3)	0 (0.0)
Stent thrombosis	19 (1.4)	10 (1.9)	2 (0.7)	4 (1.0)	3 (2.2)
Revascularization	58 (4.3)	26 (5.0)	10 (3.3)	16 (4.0)	6 (4.3)
MACCE plus unstable angina	101 (7.4)	53 (10.1)	14 (4.6)	28 (7.1)	6 (4.3)
Unstable angina	51 (3.7)	28 (5.3)	9 (3.0)	14 (3.5)	0 (0.0)
Clinically significant bleeding events	25 (1.8)	9 (1.7)	5 (1.7)	7 (1.8)	4 (2.9)
Moderate bleeding	17 (1.2)	6 (1.1)	3 (1.0)	5 (1.3)	3 (2.2)
Severe bleeding	8 (0.6)	3 (0.6)	2 (0.7)	2 (0.5)	1 (0.7)

MACCE: Major Cardiac or Cerebrovascular Adverse Events, defined as a composite of death, myocardial infarction, stroke, urgent need for revascularization, and stent thrombosis. Bleeding events were defined according to the Bleeding Academic Research Consortium (BARC) criteria. Clinically significant bleeding events was defined as a BARC score greater than or equal to 2. BARC class 2 was considered a moderate bleeding event, and BARC class 3 or higher was considered a severe bleeding event.

**TABLE 4:** Cardiovascular and Bleeding Event Incidence within 12 Months by CYP2C19 Status and P2Y_12_ inhibitors.

MACCE	Event, n (%)	IPTW-adjusted HR (95% CI)	IPTW-adjusted P value
LOF-ticagrelor	12 (4.0)	References	
LOF-clopidogrel	41 (7.8)	2.138 (1.300, 3.515)	0.003
Non-LOF-clopidogrel	23 (5.8)	1.531 (0.864, 2.714)	0.145
Non-LOF-ticagrelor	6 (4.3)	1.184 (0.582, 2.410)	0.64
MACCE plus unstable angina			
LOF-ticagrelor	14 (4.6)	References	
LOF-clopidogrel	53 (10.1)	2.442 (1.566, 3.809)	<0.001
Non-LOF-clopidogrel	28 (7.1)	1.630 (0.975, 2.724)	0.062
Non-LOF-ticagrelor	6 (4.3)	0.988 (0.502, 1.947)	0.973
Clinically significant bleeding events			
LOF-ticagrelor	5 (1.7)	References	
LOF-clopidogrel	9 (1.7)	1.477 (0.625, 3.494)	0.374
Non-LOF-clopidogrel	7 (1.8)	1.099 (0.417, 2.897)	0.849
Non-LOF-ticagrelor	4 (2.9)	1.299 (0.450, 3.749)	0.628

Compared with the LOF-ticagrelor group, the incidence of MACCE plus unstable angina was significantly higher in the LOF-clopidogrel group (10.1 vs. 4.6%; log-rank *p* = 0.006; IPTW-adjusted HR 2.442; 95% CI 1.566–3.809). There was no significant difference in the event rate of MACCE plus unstable angina between the non-LOF-clopidogrel group and LOF-ticagrelor group or between the non-LOF-ticagrelor group and LOF-ticagrelor group (Figure 2B and Table 4). We also compared clopidogrel vs. ticagrelor for the incidence of MACCE plus unstable angina in IMs and PMs. Among IMs, patients who were prescribed clopidogrel had a higher risk of developing MACCE than those who were prescribed ticagrelor (9.5 vs. 4.8%; log-rank *p* = 0.033) (Supplementary Figure S3). In PMs, clopidogrel was associated with a significantly higher incidence of MACCE than ticagrelor (event rate: 12.9 vs. 4.1%; log-rank *p* = 0.050) (Supplementary Figure S4).

During the 1-year follow-up, 25 patients suffered clinically significant bleeding events (BARC ≥2). There was no significant difference in the incidence of clinically significant bleeding events across the four groups, even after distinguishing between severe and moderate bleeding events (Figure 2C and Tables 3, 4).

After further adjustment of unbalanced covariates between the IPTW-adjusted groups, the HRs of the endpoints remained stable (Supplementary Table S5).

## Discussion

In this large single-center observational cohort study, we investigated the effect of CYP2C19 genotype-guided antiplatelet therapy on P2Y_12_ inhibitor selection and clinical outcomes. The significant findings were as follows: 1) efficient returned CYP2C19 genotype results did, to a certain extent, guide cardiologists to prescribe ticagrelor for ACS in IM/PM patients; 2) for IM/PM patients, use of clopidogrel was associated with a higher risk of developing MACCE than ticagrelor; and 3) drug selection based on CYP2C19 genotype guidance did not increase the risk of significantly clinical bleeding events within 1 year in patients.

The frequency of CYP2C19 LOF alleles in the present study was 60.7%, which was similar to previous data in other East Asian populations (Xie et al., 2013; Shen et al., 2016; Wang et al., 2016) and higher than data in other populations (approximately 30% in African American and Caucasian populations) (Klein et al., 2019). Because clopidogrel remains the most widely used P2Y_12_ inhibitor after PCI, it is vital to verify the effects of CYP2C19 genotype-guided antiplatelet therapy, especially for patients in East Asia. However, there are limited data in East Asia that have focused on CYP2C19 genotype-guided antiplatelet therapy in patients with coronary heart disease. A randomized controlled trial (Xie et al., 2013) and a prospective study (Shen et al., 2016) from China confirmed that personalized antiplatelet therapy based on CYP2C19 genotype after PCI could decrease the rates of cardiovascular adverse events with no difference in bleeding in Chinese populations. A small sample-size prospective study in Japan reported that fewer MACCE and hemorrhagic events were observed compared with conventional treatments after genotype-guided antiplatelet therapy was implemented in patients with ACS (Ozawa et al., 2018). Our real-world data support the above-mentioned studies, and demonstrate clinical benefits of genotype-guided antiplatelet therapy.

Our conclusions are also consistent with previous studies conducted in other populations. A multicenter study conducted by the IGNITE network in the United States examined the outcomes of genotype-guided P2Y_12_-receptor inhibitor therapy after PCI, and prasugrel/ticagrelor were recommended for IM/PM patients. These retrospective data proved that clopidogrel, as a maintenance treatment, had a higher MACCE risk for IM/PM patients than prasugrel or ticagrelor, especially for those with ACS indications (Cavallari et al., 2018b). Meanwhile, a single-center observational study from the United States, including 1,193 post-PCI patients demonstrated that IM/PM patients receiving prasugrel/ticagrelor had a lower risk of developing cardiovascular adverse events than those receiving clopidogrel, and no increased risk of clinically significant bleeding events was observed (Lee et al., 2018). Recently, the Patient Outcome after Primary PCI (POPular Genetics) trial in the The Netherlands involving 2,751 patients with STEMI who underwent PCI showed that genotype-guided therapy was not inferior to standard treatment with prasugrel or ticagrelor in terms of major cardiovascular adverse events and had a lower incidence of bleeding risk events (Claassens et al., 2019). The Pharmacogenetics of Clopidogrel in Acute Coronary Syndromes (PHARMCLO) trial in Italy also demonstrated that composite endpoints of ischemic events and major bleeding events were reduced in the genotype-guided group compared with the conventional treatment group (Notarangelo et al., 2018).

In this non-intervention study, 36.6% of patients with a LOF allele were prescribed ticagrelor, which was lower that the corresponding proportions of 60.5% in the IGNITE study (Cavallari et al., 2018b) and 53% in another study (Tuteja et al., 2020). Although important, the CYP2C19 genotype is not the only factor considered when prescribing P2Y_12_ inhibitors. Various clinical factors, especially risk factors for bleeding, are related to the use of clopidogrel. Some of these potential factors also make cardiologists very cautious about prescribing ticagrelor. First, previous reports indicated that Asian ACS patients had a higher risk of drug-related bleeding and a lower risk of ischemia than Caucasian ACS patients (Mak et al., 2009; Kumar et al., 2013; Levine et al., 2014). Second, ticagrelor had higher discontinuation rates and costs than clopidogrel (Wallentin et al., 2009; Bonaca et al., 2015). These concerns may stop clinicians from prescribing ticagrelor even after receiving an unoptimistic pharmacogenomics result. However, given the higher risk of ischemia associated with clopidogrel use in patients with a LOF allele, and the clear cardiovascular benefits of using ticagrelor, it is recommended that clinicians should increase the weight of genetics when choosing P2Y_12_ inhibitors.

We observed an imbalance between IMs (44.0%) and PMs (34.7%) when physicians implemented genotype-guided drug selection. Such inequalities also existed in other previous studies (Cavallari et al., 2018b; Tuteja et al., 2020). Controversy about the value of IM status in genotype-guided antiplatelet therapy has always existed, despite sufficient evidence that IMs are associated with reduced clinical efficacy of clopidogrel (Xi et al., 2019). Previously, the CPIC recommended an alternative therapy for IM patients (Scott et al., 2013). However, in the clopidogrel drug manual, the black box warning from the FDA only recommends that PMs should receive P2Y_12_ receptor inhibitors other than clopidogrel, and does not mention the risk for IMs (Holmes et al., 2010). Our real-world data prove that an increased risk of cardiovascular adverse events exists in both PMs and IMs. This conclusion not only validates the results of the IGNITE study (Cavallari et al., 2018b) and earlier studies (Xie et al., 2013; Shen et al., 2016; Gross et al., 2018; Lee et al., 2018; Notarangelo et al., 2018; Claassens et al., 2019; Khan et al., 2019), but also supports recommendations for prescribing alternative therapies to IMs and PMs from the CPIC (Scott et al., 2013). Therefore, we recommend that cardiologists should pay attention to the risk of cardiovascular adverse events in IMs, and increase the weight of IMs when making drug selection decisions, similar to the case for PMs.

Improving the adoption of genetic testing results is very urgent and challenging. In our study, although all of the genotype results were available before the patients were discharged, our data and those in previous studies (Bagai et al., 2014; Lee et al., 2018; Tuteja et al., 2020) suggested that clinicians were reluctant to switch existing treatments regardless of genetic outcomes. It is necessary to provide feedback on the results of pharmacogenomics tests as quickly as possible, because this can help physicians to adopt CYP2C19 genotype results. We agree that delayed return of genetic test reports may result in lower acceptance of the genetic results (Cavallari et al., 2018a; Tuteja et al., 2020). Regular conduct of clinician education is also essential, because it allows cardiologists to continue to pay attention to pharmacogenomics reports (Bell et al., 2014). Besides, the participation of clinical pharmacists can support the clinical decision-making of cardiologists, which is very important (Owusu-Obeng et al., 2014).

Based on our real-world data, clopidogrel was similar to ticagrelor in reducing cardiovascular clinical events in patients without a LOF allele. Because prescription of clopidogrel has better safety and economic profiles, it is worth encouraging the prescription of clopidogrel for NM/RM/UM patients. For patients prescribed ticagrelor after PCI, early de-escalation to clopidogrel may be a strategy worth considering. The results of The TROPICAL-ACS genotyping substudy (Gross et al., 2018) and a recent observational study (Martin et al., 2020) confirmed that de-escalation to clopidogrel in UM/RM/NM patients did not significantly increase the risk of MACCE compared with continuation of ticagrelor/prasugrel. However, there is very little clinical evidence from the East Asian population to verify this strategy, and this will be the focus of our future studies.

Our study has several limitations. First, due to the inherent limitations of observational studies, the implementation of genotype-guided drug therapy in this study was non-randomized. Therefore, whether to detect genotypes and whether to obey genotype guidance were dependent on the decisions of individual doctors. Although we tried to balance the covariates at baseline by IPTW adjustment, we were still unable to rule out residual confounding. Second, the proportions of STEMI and non-STEMI were relatively low in real-world ACS patients who received genotype testing, which is inconsistent with epidemiological data (Wang et al., 2020). The reason may be that some patients with myocardial infarction were prescribed ticagrelor upon admission, and their genetic testing was unnecessary. However, in the Supplementary Material, we have demonstrated the clinical benefits of gene-guided drug selection in both STEMI/non-STEMI and UA. Therefore, the conclusions of this study are robust. Third, patients who did not have CYP2C19 genotype results were not included in the study. Therefore, we did not explore the factors that influence clinicians’ indications and frequencies of CYP2C19 genotype testing. Fourth, since prasugrel is not licensed in China, ticagrelor was the only P2Y_12_ receptor inhibitor used for alternative therapy. However, the results of a previous meta-analysis suggested that ticagrelor and prasugrel showed no difference in preventing adverse cardiovascular events and bleeding events in patients with ACS (Khan et al., 2019). Fifth, the conclusions of the study were based on a registration database from a single medical center in China, and thus the results may not be widely applicable to other settings or populations.

In conclusion, this observational study in a real-world setting confirmed that CYP2C19 genomics test results could guide cardiologists to prescribe ticagrelor for IM/PM patients, but this is not universal. In patients with a LOF allele, the incidence of MACCE with clopidogrel was significantly higher than that with ticagrelor. The findings of the present study support the implementation of CYP2C19 gene-guided antiplatelet therapy in clinical practice. However, our conclusions still need to be proven by large multicenter randomized controlled trials.

## Data Availability Statement

The raw data supporting the conclusions of this article will be made available by the authors, without undue reservation, to any qualified researcher.

## Ethics Statement

The studies involving human participants were reviewed and approved by the Ethics Committee of the Clinical Research Center at Beijing Anzhen Hospital. The patients/participants provided their written informed consent to participate in this study.

## Author Contributions

Conception and design: YZ and YL. Administrative support: YL and X-JS. Determination of clinical events: S-WC, NN, and YZ. Collection and upload of data: YZ, W-XP, J-LH, B-DL, RZ, Y-NZ, J-LY, J-JW, Y-FW, and Z-WF. Data analysis and interpretation: YZ, J-LH, and B-DL. Manuscript writing: All authors. Final approval of manuscript: All authors.

## Funding

This study was supported by the National Major Scientific and Technological Special Project for “Significant New Drugs Development” during the Thirteenth Five-year Plan Period (2017ZX09304017) and Beijing Municipal Administration of Hospitals Clinical Medicine Development of Special Funding Support (Grant Number ZYLX201805).

## Conflict of Interest

The authors declare that the research was conducted in the absence of any commercial or financial relationships that could be construed as a potential conflict of interest.
