# Supplementary material for: Impact of Implementing CYP2C19 Genotype-Guided Antiplatelet Therapy on P2Y12 Inhibitor Selection and Clinical Outcomes in Acute Coronary Syndrome Patients After Percutaneous Coronary Intervention: A Real-World Study in China
Source: Front Pharmacol. 2021 Jan 20;11:582929. doi: 10.3389/fphar.2020.582929 (PMC7854467; doi:10.3389/fphar.2020.582929)
Supplement: Supplementary file 1 [file table1.docx]

**Table S1.** The Factors Affecting the Cardiologist's P2Y_12_ Receptor Inhibitor Selection at Discharge.

|  | **Total** | **Univariate regression analysis** | | **Multivariate regression analysis** | |
| --- | --- | --- | --- | --- | --- |
|  | **n=1631 (%)** | **OR (95%Cl)** | **P value** | **Adjusted OR (95%Cl)** | **P value** |
| with a LOF alles | 826 (60.7) | 1.658 (1.304, 2.108) | ＜0.001 | 1.349 (1.040, 1.751) | 0.024 |
| Age | 60.161 ± 9.733 | 0.976 (0.965, 0.988) | ＜0.001 | 0.984 (0.970, 0.997) | 0.018 |
| Female | 331 (24.3) | 0.769 (0.586, 1.010) | 0.059 |  |  |
| Minorities. | 27 (2.0) | 1.972 (0.919, 4.233) | 0.081 |  |  |
| BMI, kg/m^2^ | 25.826 ± 3.291 | 1.024 (0.989, 1.061) | 0.174 |  |  |
| Current smoker | 303 (22.3) | 1.102 (0.841, 1.444) | 0.482 |  |  |
| indexed PCI for UA | 1185 (87.1) | 0.524 (0.380, 0.722) | ＜0.001 | 0.584 (0.404, 0.844) | 0.004 |
| No. of stent, n | 1.607 ± 1.041 | 1.261 (1.133, 1.404) | ＜0.001 | 1.261 (1.119, 1.421) | ＜0.001 |
| Stent implantation* | 1256 (92.3) | 2.629 (1.544, 4.476) | ＜0.001 | 2.515 (1.414, 4.473) | 0.002 |
| Clopidogrel use before admission | 331 (24.3) | 0.631 (0.477, 0.835) | 0.001 | 0.591 (0.437, 0.800) | ＜0.001 |
| Ticagrelor use before admission | 36 (2.6) | 13.886 (5.361, 35.967) | ＜0.001 | 16.850 (5.845, 48.573) | ＜0.001 |
| Previous PCI with stenting | 434 (31.9) | 1.011 (0.792, 1.289) | 0.931 |  |  |
| Previous CABG | 54 (4.0) | 0.877 (0.483, 1.591) | 0.665 |  |  |
| Hypertension | 865 (63.6) | 0.703 (0.556, 0.887) | 0.003 | 0.673 (0.519, 0.872) | 0.003 |
| Hyperlipidemia | 712 (52.3) | 1.126 (0.897, 1.414) | 0.306 |  |  |
| Atrial fibrillation | 35 (2.6) | 0.263 (0.092, 0.751) | 0.013 |  |  |
| Myocardial infarction | 184 (13.5) | 1.201 (0.867, 1.663) | 0.27 |  |  |
| Diabetes mellitus | 470 (34.5) | 1.078 (0.849, 1.367) | 0.538 |  |  |
| End-stage kidney disease | 18 (1.3) | 0.594 (0.194, 1.816) | 0.361 |  |  |
| Heart failure | 16 (1.2) | 0.479 (0.136, 1.691) | 0.253 |  |  |
| Cerebral infarction | 97 (7.1) | 0.710 (0.444, 1.136) | 0.154 |  |  |
| Gastrointestinal bleed | 3 (0.2) | 0.000 (0.000, inf.) | 0.967 |  |  |
| Left ventricular EF% | 62.783 ± 7.311 | 0.980 (0.965, 0.997) | 0.017 |  |  |
| eGFR | 0.572 ± 0.133 | 1.577 (0.667, 3.728) | 0.299 |  |  |

Values are mean ± SD or n (percentage); OR: odds ratio

LOF: loss-of-function; BMI: body mass index; PCI: percutaneous coronary intervention; CABG: coronary-artery bypass grafting; EF: ejection fraction; eGFR: estimate glomerular filtration rate.

*Stent implantation: All implanted stents were drug-eluting stents.
