# Supplementary material for: Impact of Implementing CYP2C19 Genotype-Guided Antiplatelet Therapy on P2Y12 Inhibitor Selection and Clinical Outcomes in Acute Coronary Syndrome Patients After Percutaneous Coronary Intervention: A Real-World Study in China
Source: Front Pharmacol. 2021 Jan 20;11:582929. doi: 10.3389/fphar.2020.582929 (PMC7854467; doi:10.3389/fphar.2020.582929)
Supplement: Supplementary file 2 [file table2.docx]

**Table S2.** Patient Baseline Demographic and Clinical Characteristics of Non-LOF-Clopidogrel and Non-LOF-Ticagrelor Before and After Adjustment With IPTW.

|  | **Non-LOF-Clopidogrel** | **Non-LOF-Ticagrelor** | **Standardized Difference** | **Non-LOF-Clopidogrel** | **Non-LOF-Ticagrelor** | **IPTW-adjusted Standardized Difference** |
| --- | --- | --- | --- | --- | --- | --- |
|  | **n=397** | **n=138** |  | **n=397** | **n=138** |  |
| Age | 60.78 ± 9.50 | 58.70 ± 9.72 | 0.22 | 60.09 ± 10.03 | 60.09 ± 9.15 | 0 |
| Female | 97 (24.4) | 28 (20.3) | 0.1 | 94(23.6) | 31(22.4) | -0.03 |
| Han | 392 (98.7) | 133 (96.4) | 0.15 | 389(98.0) | 135(97.8) | -0.02 |
| Body mass index | 25.85 ± 3.24 | 25.67 ± 3.18 | 0.06 | 25.79 ± 3.06 | 26.07 ± 3.39 | 0.09 |
| Current smoker new | 90 (22.7) | 25 (18.1) | 0.11 | 84(21.2) | 32(22.9) | 0.04 |
| **No of stent** |  |  |  |  |  |  |
| <1 | 40 (10.1) | 4 (2.9) | 0.29 | 31(7.9) | 10(7.3) | -0.02 |
| ≥1, <3 | 297 (74.8) | 106 (76.8) | 0.05 | 301(75.7) | 106(77.1) | 0.03 |
| ≥3 | 60 (15.1) | 28 (20.3) | 0.14 | 65(16.4) | 21(15.5) | -0.02 |
| **PCI indicatin** |  |  |  |  |  |  |
| STEMI | 15 (3.8) | 14 (10.1) | 0.25 | 20(5.1) | 7(5.0) | -0.01 |
| Non-STEMI | 23 (5.8) | 12 (8.7) | 0.11 | 27(6.8) | 11(7.8) | 0.04 |
| **P2Y_12_ inhibitor use before admission** |  |  |  |  |  |  |
| Clopidogrel | 99 (24.9) | 30 (21.7) | 0.08 | 97(24.5) | 34(24.7) | 0 |
| Ticagrelor | 2 (0.5) | 11 (8.0) | 0.38 | 3(0.83) | 3(2.4) | **0.12** |
| Previous PCI with stenting | 122 (30.7) | 41 (29.7) | 0.02 | 126(31.8) | 45(32.7) | 0.02 |
| Previous CABG | 18 (4.5) | 4 (2.9) | 0.09 | 15(3.7) | 6(4.2) | 0.02 |
| Hypertension | 260 (65.5) | 85 (61.6) | 0.08 | 253(63.7) | 88(63.5) | 0 |
| Hyperlipidemia | 207 (52.1) | 79 (57.3) | 0.1 | 218(54.9) | 78(56.5) | 0.03 |
| Diabetes mellitus | 130 (32.8) | 60 (43.5) | 0.22 | 139(34.9) | 49(35.8) | 0.02 |
| Myocardial infarction | 56 (14.1) | 20 (14.5) | 0.01 | 61(15.4) | 22(16.0) | 0.02 |
| Atrial fibrillation | 15 (3.8) | 2 (1.5) | 0.15 | 14(3.7) | 2(1.5) | **-0.14** |
| Gastrointestinal bleed | 1 (0.3) | 0 (0.0) | 0.07 | 1(0.2) | 0(0.0) | -0.07 |
| End-stage kidney disease | 4 (1.0) | 3 (2.2) | 0.09 | 6(1.5) | 2(1.3) | -0.02 |
| Heart failure | 4 (1.0) | 1 (0.7) | 0.03 | 4(0.9) | 0(0.3) | -0.08 |
| Cerebral infarction | 26 (6.6) | 7 (5.1) | 0.06 | 23(5.8) | 7(5.2) | -0.03 |
| Left ventricular EF% | 63.06 ± 6.98 | 61.95 ± 6.84 | 0.16 | 62.73 ± 7.30 | 62.70 ± 7.06 | 0 |
| eGFR | 0.57 ± 0.12 | 0.59 ± 0.14 | 0.18 | 0.57 ± 0.12 | 0.57 ± 0.14 | 0 |
| aspirin | 397 (100.0) | 138(100.0) | - | 397 (100.0) | 138(100.0) | - |
| Anticoagulant agent | 3 (0.8) | 0 (0.0) | 0.12 | 3(0.7) | 0(0.0) | **-0.12** |
| Statin | 394 (99.2) | 137 (99.3) | 0 | 394(99.1) | 137(99.2) | 0.01 |
| ACEI or ARB | 139 (35.0) | 46 (33.3) | 0.04 | 136(34.3) | 45(32.5) | -0.04 |
| Beta blocker | 263 (66.3) | 89 (64.5) | 0.04 | 264(66.4) | 95(68.5) | 0.05 |
| Proton pump inhibitor | 349 (87.9) | 121 (87.7) | 0.01 | 349(87.9) | 122(88.5) | 0.02 |
| Morisky score ≥6 | 383 (96.5) | 131 (94.9) | 0.08 | 381(95.9) | 133(96.6) | 0.04 |

Values are mean ± SD or n (percentage);

IPTW: Inverse Probability of Treatment Weights

If the Standardized Difference ≥ 0.1, it indicates that the variable is not well balanced between groups after adjusting IPTW.
