# Supplementary material for: Impact of Implementing CYP2C19 Genotype-Guided Antiplatelet Therapy on P2Y12 Inhibitor Selection and Clinical Outcomes in Acute Coronary Syndrome Patients After Percutaneous Coronary Intervention: A Real-World Study in China
Source: Front Pharmacol. 2021 Jan 20;11:582929. doi: 10.3389/fphar.2020.582929 (PMC7854467; doi:10.3389/fphar.2020.582929)
Supplement: Supplementary file 3 [file table3.docx]

**Table S3.** Cardiovascular and Bleeding Event Incidence Between Non-LOF-Clopidogrel and Non-LOF-Ticagrelor.

| **Endpoint** | **Non-LOF-Ticagrelor** | **Non-LOF-Clopidogrel** | **Adjusted IPTW HR (95% CI)** | **P Value** | **Adjusted IPTW HR* (95% CI)** | **P Value*** |
| --- | --- | --- | --- | --- | --- | --- |
| **MACCE** | 6 (4.3) | 23 (5.8) | 1.055 (0.586, 1.902) | 0.858 | 0.997 (0.549, 1.810) | 0.99291 |
| **MACCE plus Unstable Angina** | 6 (4.3) | 28 (7.1) | 1.367 (0.784, 2.383) | 0.270 | 1.309 (0.747, 2.292) | 0.34695 |
| **Clinically Significant Bleeding Events** | 4 (2.9) | 7 (1.8) | 0.492 (0.192, 1.261) | 0.140 | 0.495 (0.193, 1.269) | 0.143 |

CI indicates confidence interval; HR, hazard ratio; LOF: loss-of-function; MACCE: major adverse cardiovascular and cerebrovascular events; Clinically Significant Bleeding Events: BARC score greater than or equal to 2.

*adjusted for covariates that were not optimally balanced after IPTW (standard deviation ≥0.10)
