# Supplementary material for: Impact of Implementing CYP2C19 Genotype-Guided Antiplatelet Therapy on P2Y12 Inhibitor Selection and Clinical Outcomes in Acute Coronary Syndrome Patients After Percutaneous Coronary Intervention: A Real-World Study in China
Source: Front Pharmacol. 2021 Jan 20;11:582929. doi: 10.3389/fphar.2020.582929 (PMC7854467; doi:10.3389/fphar.2020.582929)
Supplement: Supplementary file 4 [file table4.docx]

**Table S4.** The Incidence of Clinical Endpoints Between Clopidogrel and Ticagrelor Was Stratified by The Indications for PCI In Patients with A Loss-Of-Function Allele.

|  | STEMI/Non-STEMI | | | | Unstable Angina | | | |
| --- | --- | --- | --- | --- | --- | --- | --- | --- |
|  | LOF-Clopidogrel n=58 | LOF-Ticagrelor n=54 | IPTW-adjusted HR (95 CI) for LOF-Clopidogrel vs. LOF-Ticagrelor | P value | LOF-Clopidogrel n=466 | LOF-Ticagrelor n=248 | IPTW-adjusted HR (95 CI) for LOF-Clopidogrel vs. LOF-Ticagrelor | P value |
| MACCE | 6 (10.3) | 1 (1.9) | 2.942 (0.617, 14.034) | 0.176 | 35 (7.5) | 11 (4.4) | 2.051 (1.213, 3.467) | 0.007 |
| MACCE plus Unstable Angina | 7 (12.1) | 1 (1.9) | 4.289 (0.960, 19.164) | 0.056 | 46 (9.9) | 13 (5.2) | 2.282 (1.431, 3.641) | <0.001 |
| Clinically significant bleeding events | 1 (1.7) | 2 (3.7) | 0.804 (0.175, 3.693) | 0.779 | 8 (1.7) | 3 (1.2) | 2.001 (0.679, 5.897) | 0.208 |

CI indicates confidence interval; HR, hazard ratio; STEMI: ST-segment elevation myocardial infarction; Non-STEMI: non-ST-segment elevation myocardial infarction
