# Supplementary material for: Impact of Implementing CYP2C19 Genotype-Guided Antiplatelet Therapy on P2Y12 Inhibitor Selection and Clinical Outcomes in Acute Coronary Syndrome Patients After Percutaneous Coronary Intervention: A Real-World Study in China
Source: Front Pharmacol. 2021 Jan 20;11:582929. doi: 10.3389/fphar.2020.582929 (PMC7854467; doi:10.3389/fphar.2020.582929)
Supplement: Supplementary file 5 [file table5.docx]

**Table S5.** Clinical Outcomes between Groups after Adjusting for Covariates That Were Not Optimally Balanced After IPTW (Standard Deviation ≥0.10).

| **Endpoint** | LOF-Clopidogrel | LOF-Ticagrelor | Non-LOF-Clopidogrel | Non-LOF-Ticagrelor | LOF-Clopidogrel vs. LOF-Ticagrelor Adjusted IPTW HR (95 CI), *p* value | Non-LOF-Clopidogrel vs. LOF-Ticagrelor Adjusted IPTW HR (95 CI), *p* value | Non-LOF-Ticagrelor vs. LOF-Ticagrelor Adjusted IPTW HR (95 CI), *p* value |
| --- | --- | --- | --- | --- | --- | --- | --- |
| **MACCE** | 41 (7.8) | 12 (4.0) | 23 (5.8) | 6 (4.3) | 2.084 (1.263, 3.438), 0.004 | 1.495 (0.839, 2.666), 0.173 | 1.194 (0.587, 2.429), 0.625 |
| **MACCE plus Unstable Angina** | 53 (10.1) | 14 (4.6) | 28 (7.1) | 6 (4.3) | 2.369 (1.514, 3.706), <0.001 | 1.598 (0.952, 2.680), 0.076 | 0.996 (0.506, 1.963), 0.991 |
| **Clinically Significant Bleeding Events** | 9 (1.7) | 5 (1.7) | 7 (1.8) | 4 (2.9) | 1.504 (0.636, 3.557), 0.353 | 1.087 (0.412, 2.866), 0.867 | 1.309 (0.454, 3.778), 0.618 |
