# Supplementary figures and images for: Impact of Implementing CYP2C19 Genotype-Guided Antiplatelet Therapy on P2Y12 Inhibitor Selection and Clinical Outcomes in Acute Coronary Syndrome Patients After Percutaneous Coronary Intervention: A Real-World Study in China
Source: Front Pharmacol. 2021 Jan 20;11:582929. doi: 10.3389/fphar.2020.582929 (PMC7854467; doi:10.3389/fphar.2020.582929)

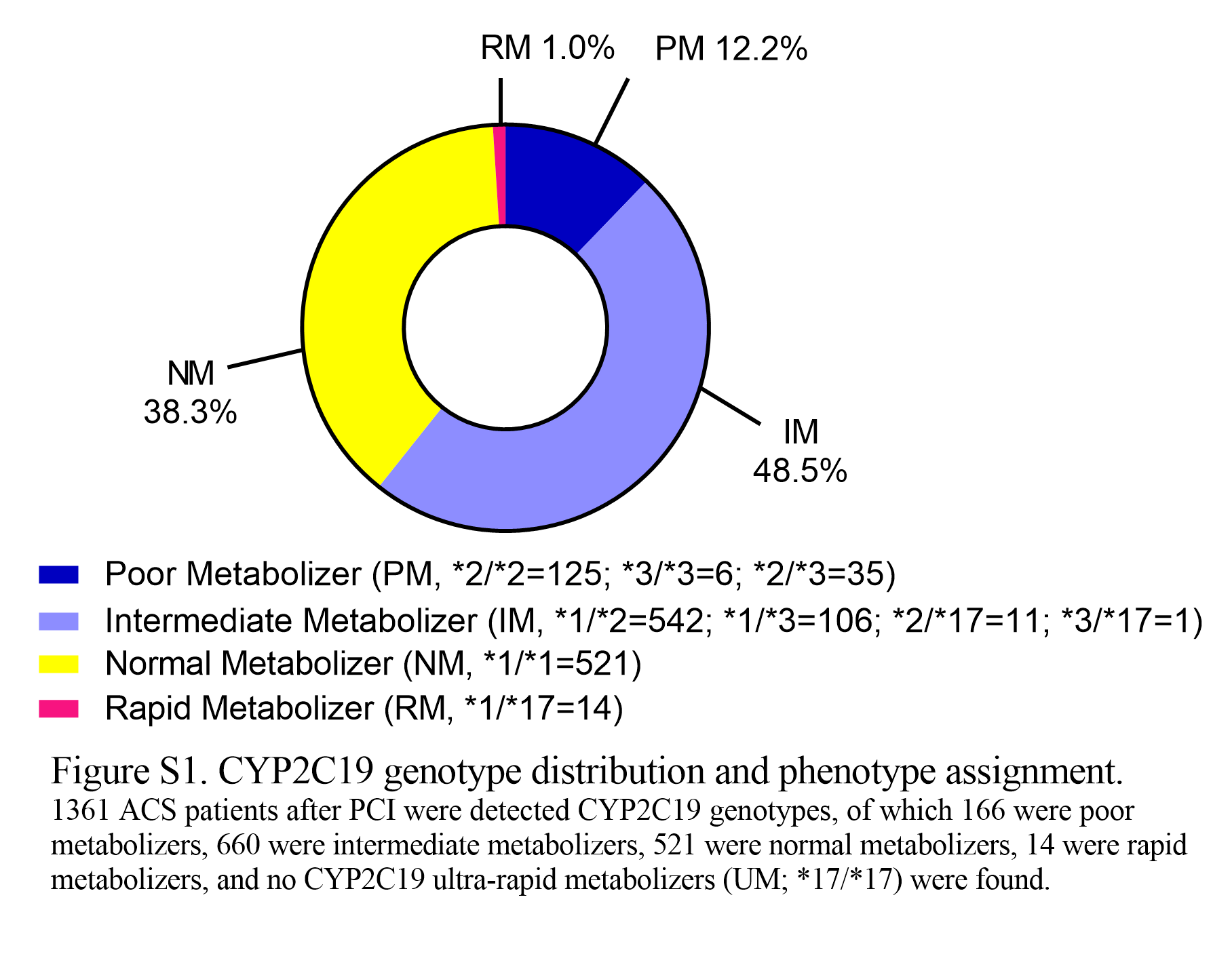

Supplement: Supplementary file 6 [file image1.tif]

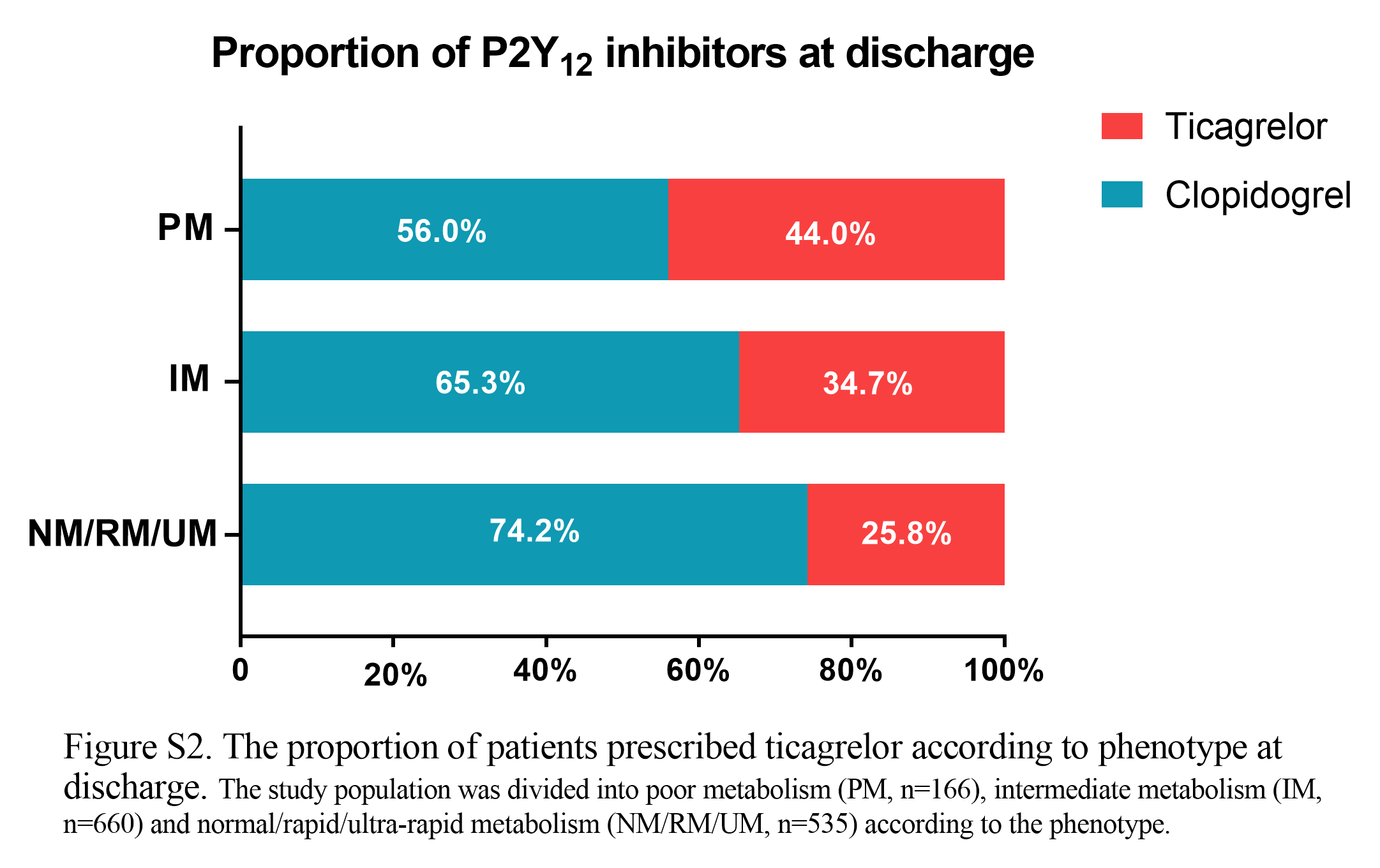

Supplement: Supplementary file 7 [file image2.tif]

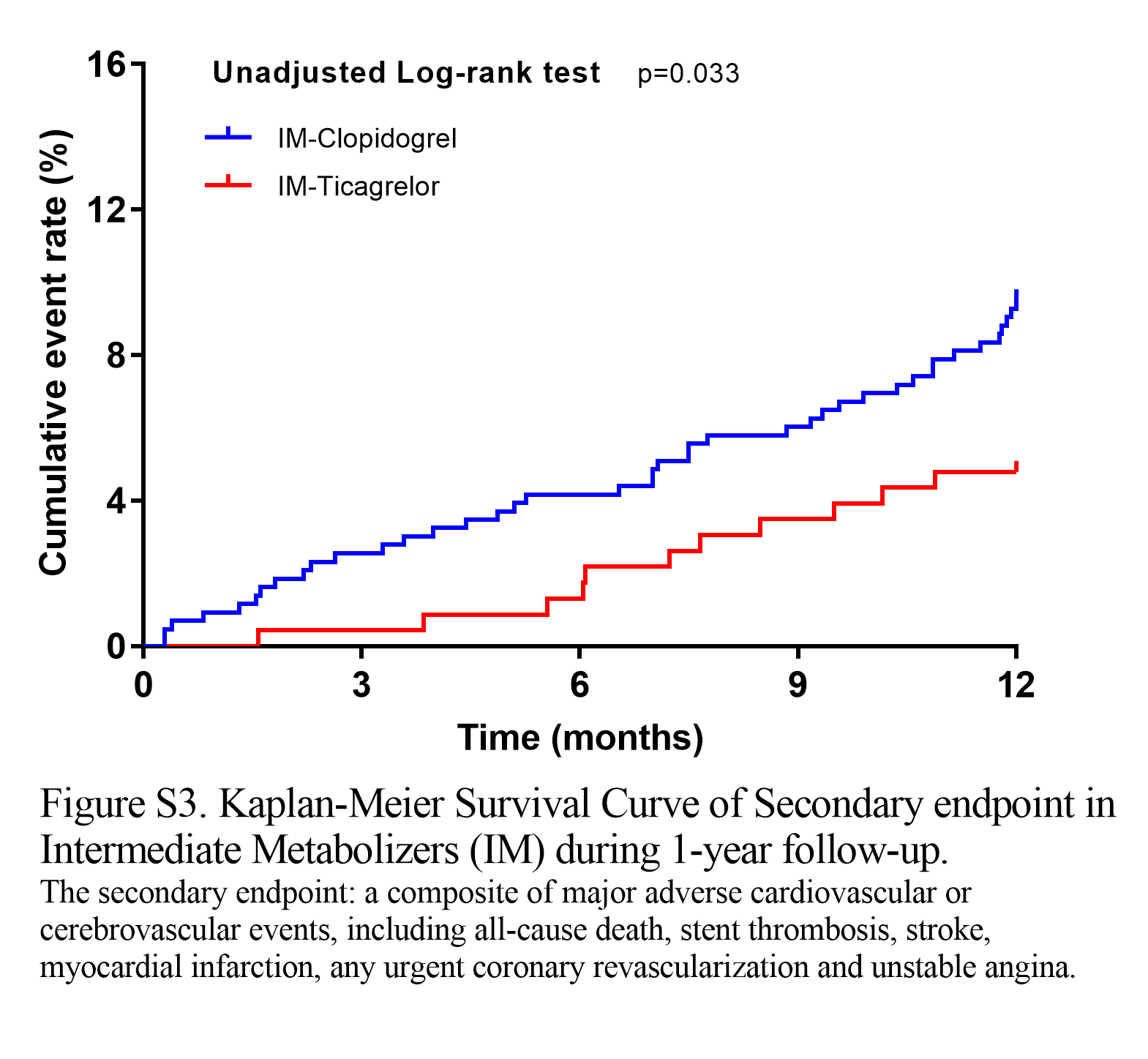

Supplement: Supplementary file 8 [file image3.tif]

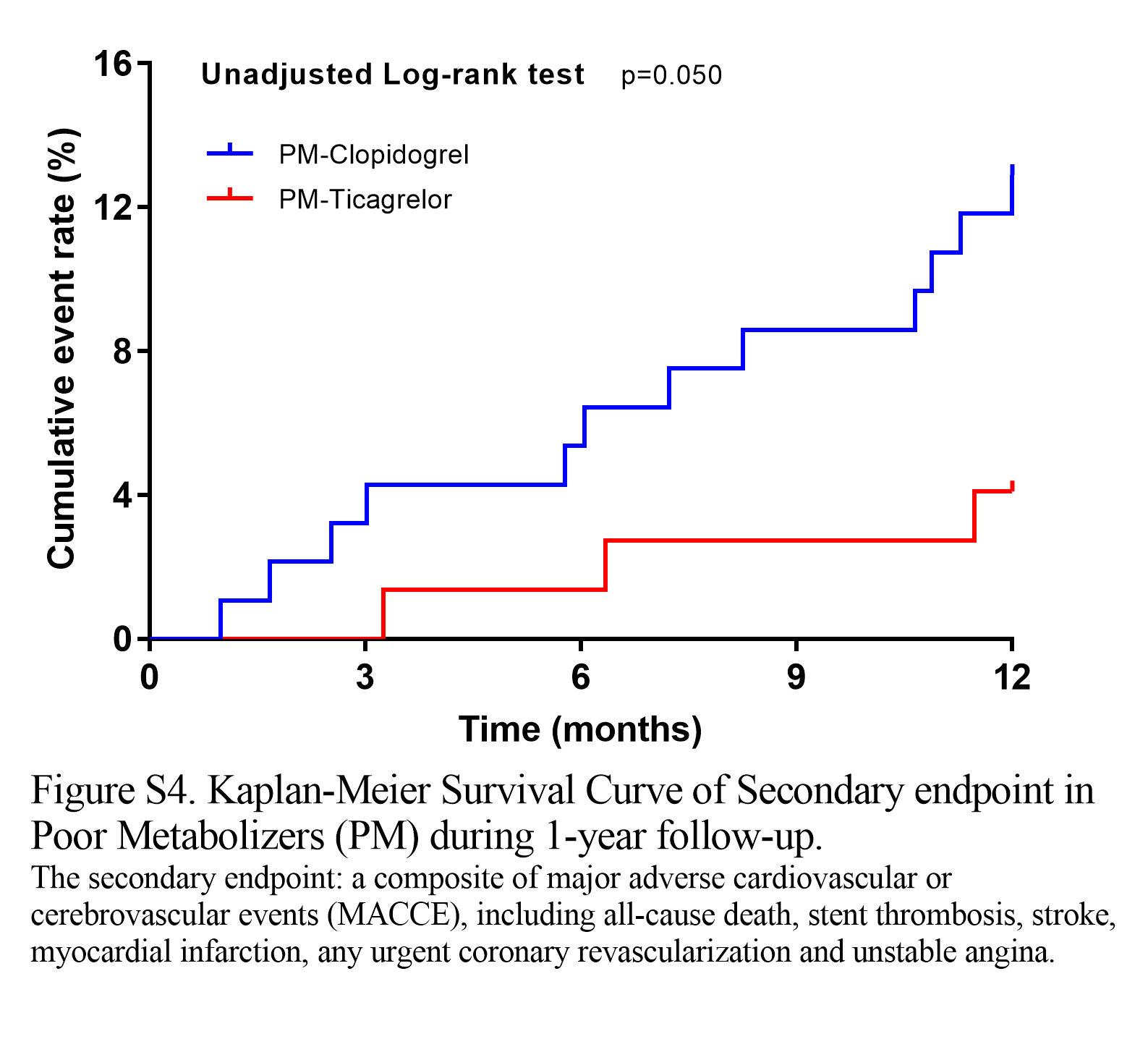

Supplement: Supplementary file 9 [file image4.tif]
